# Supplementary material for: Bactopia: a Flexible Pipeline for Complete Analysis of Bacterial Genomes
Source: mSystems. 2020 Aug 4;5(4):e00190-20. doi: 10.1128/mSystems.00190-20 (PMC7406220; doi:10.1128/mSystems.00190-20)
Supplement: DATA SET S3 [file mSystems.00190-20-sd003.pdf]

Nextflow workflow report

[cranky_torricelli]

Workflow execution completed successfully!

Run times

05-Nov-2019 08:06:30 - 07-Nov-2019 20:21:36 (duration: **2d 12h 15m 6s**)

31295 succeeded

Nextflow command

```
nextflow /home/rpetit3/repos/bactopia/main.nf --accessions ../lactobacillus-accessions.txt --datasets /home/rpetit3/datasets --species lactobacillus --coverage 100 --cpus 4 -profile slurm --min_genome_size 1000000 --max_genome_size 4200000
```

**CPU-Hours**	3'052.4 (0% failed)
**Launch directory**	/home/rpetit3/projects/lactobacillus/bactopia
**Work directory**	/home/rpetit3/projects/lactobacillus/bactopia/work
**Project directory**	/home/rpetit3/repos/bactopia
**Script name**	main.nf
**Script ID**	b763323ed40fb19eda2aff0c1a481f11
**Workflow session**	90a3fb3c-b891-4c5a-a6b5-5c6159277170
**Workflow profile**	slurm
**Nextflow version**	version 19.10.0, build 5170 (21-10-2019 15:07 UTC)

Resource Usage

These plots give an overview of the distribution of resource usage for each process.

CPU

Raw Usage | % Allocated

CPU Usage

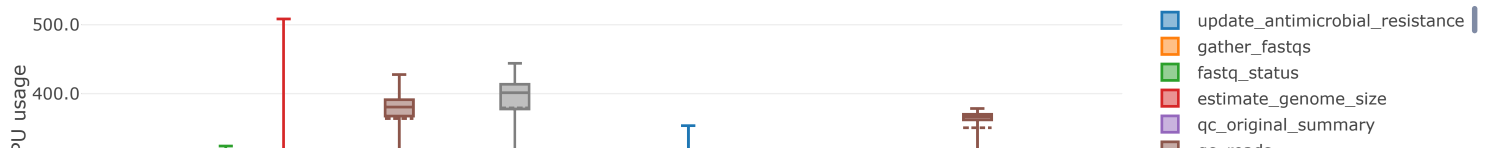

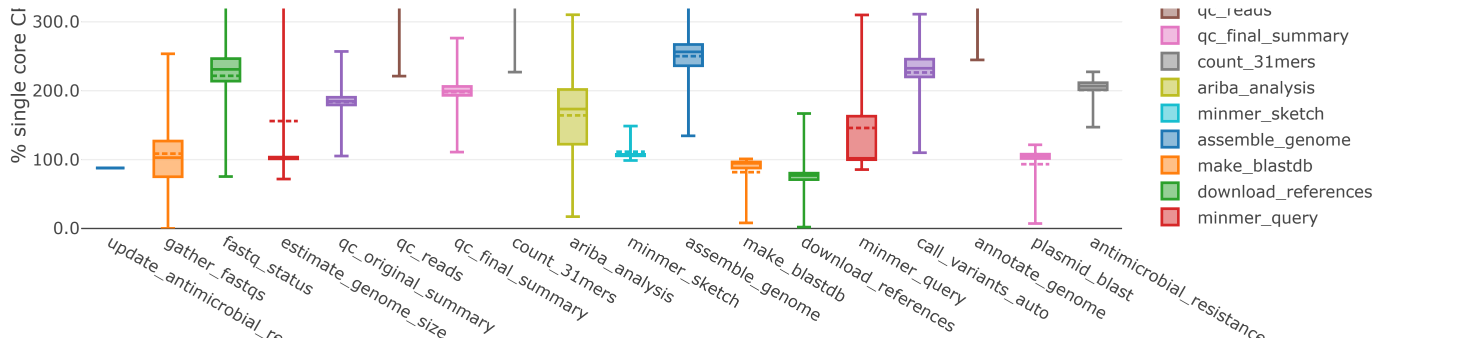

Memory

Physical (RAM) | Virtual (RAM + Disk swap) | % RAM Allocated

Physical Memory Usage

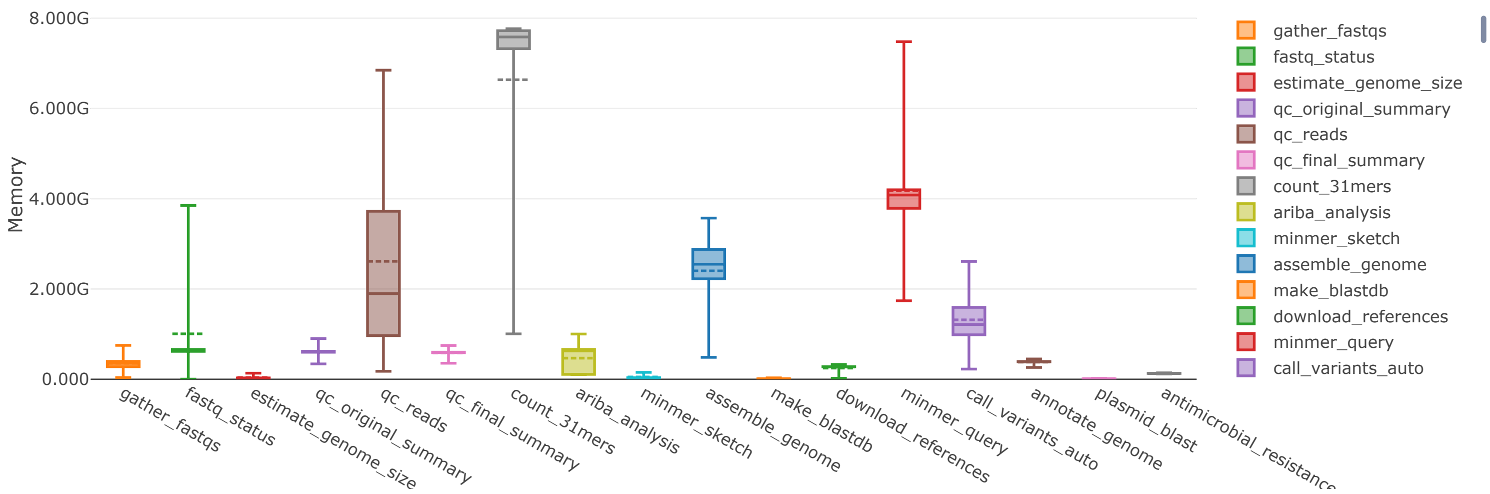

Job Duration

Raw Usage | % Allocated

Task execution real-time

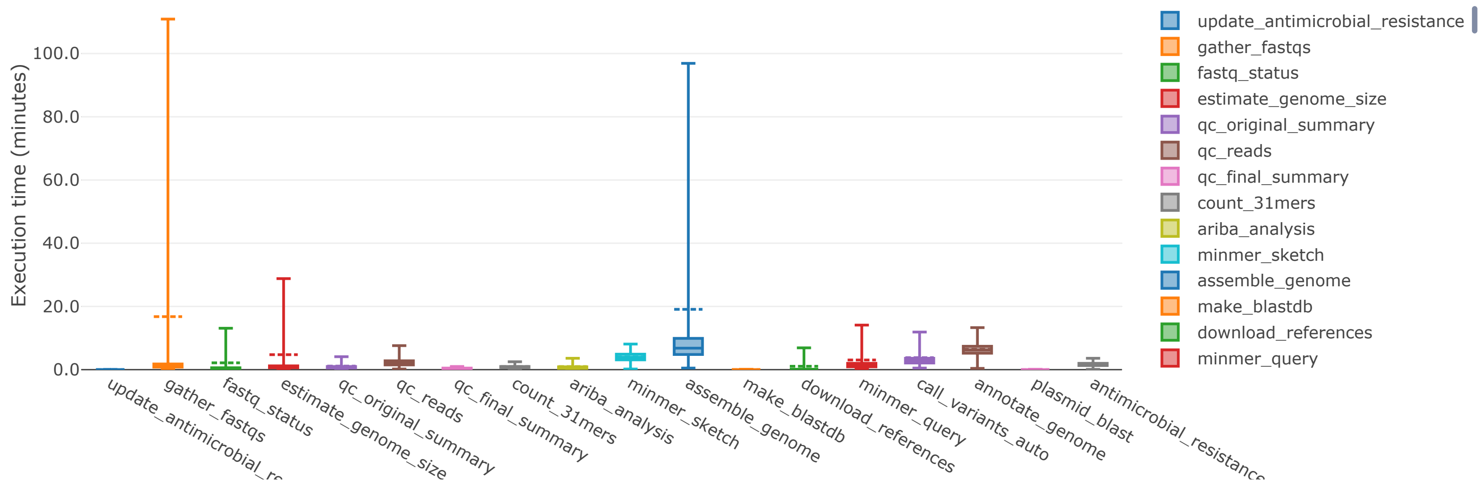

I/O

Read Write

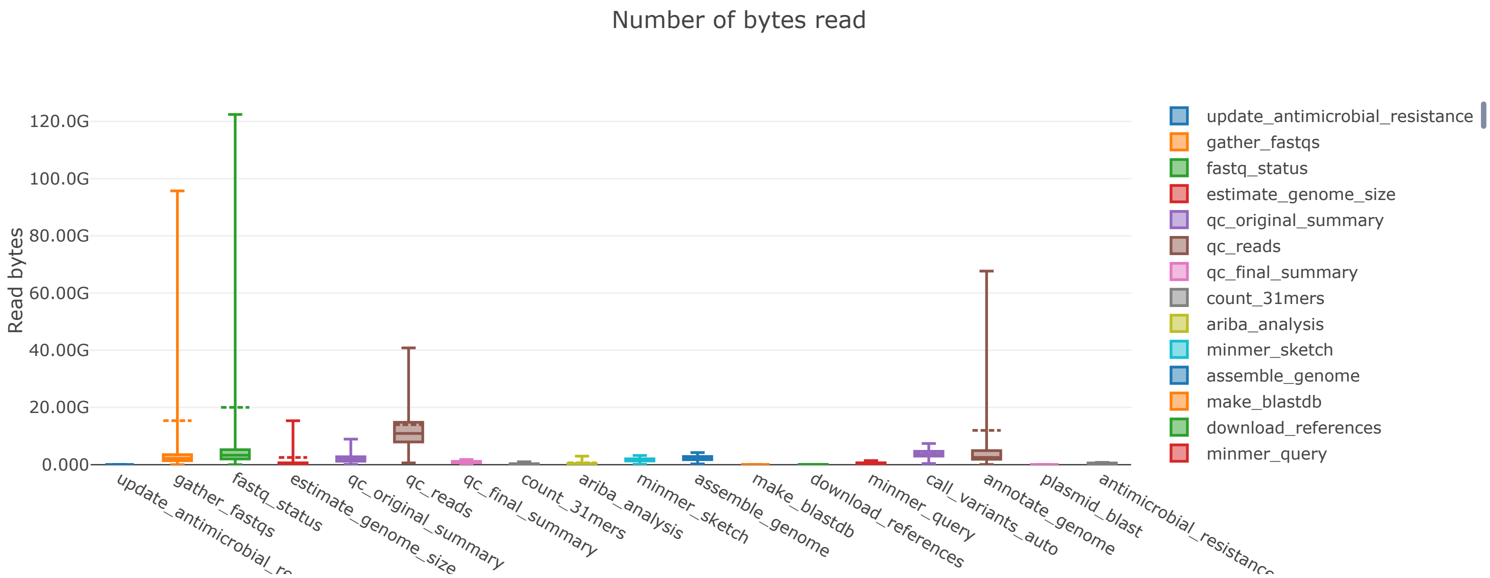

(tasks table omitted because the dataset is too big)

Generated by Nextflow, version 19.10.0
