# Supplementary figures and images for: Bactopia: a Flexible Pipeline for Complete Analysis of Bacterial Genomes
Source: mSystems. 2020 Aug 4;5(4):e00190-20. doi: 10.1128/mSystems.00190-20 (PMC7406220; doi:10.1128/mSystems.00190-20)

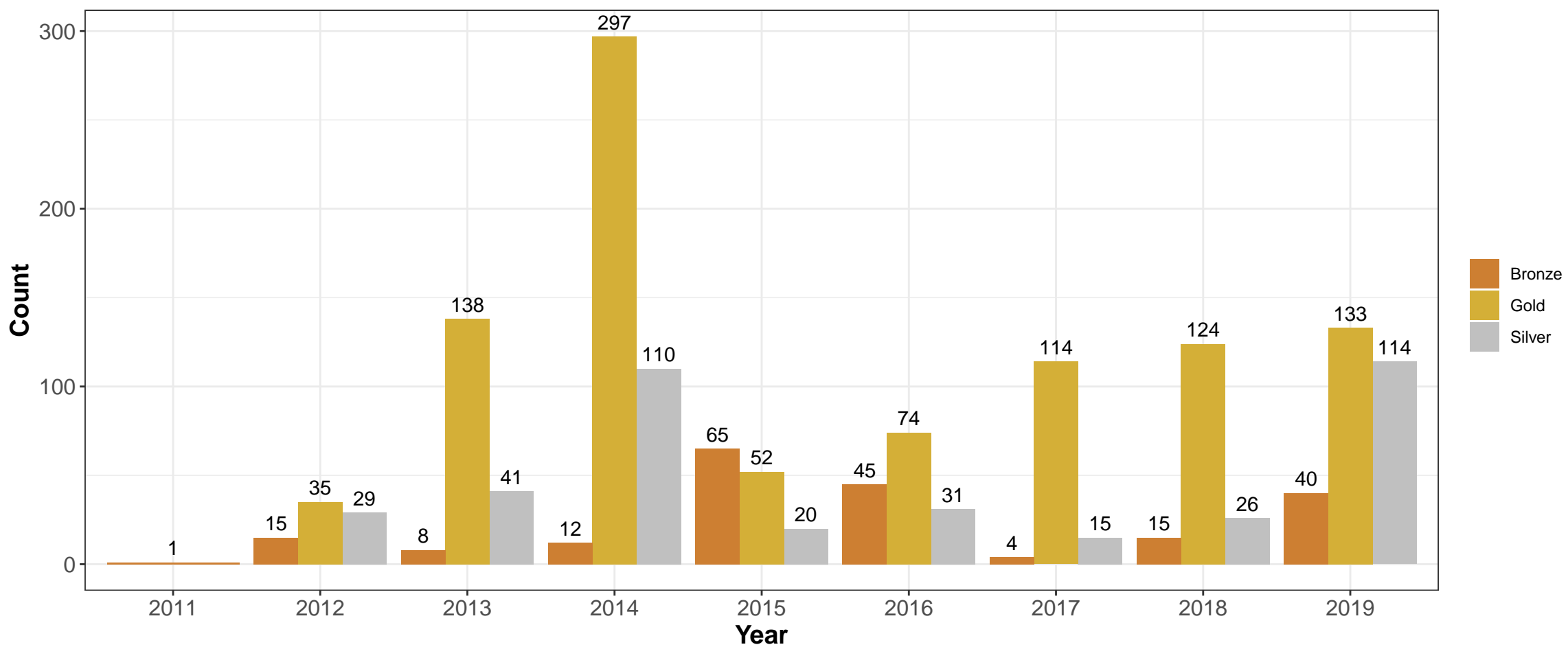

Supplement: FIG S2 [file mSystems.00190-20-sf002.pdf]

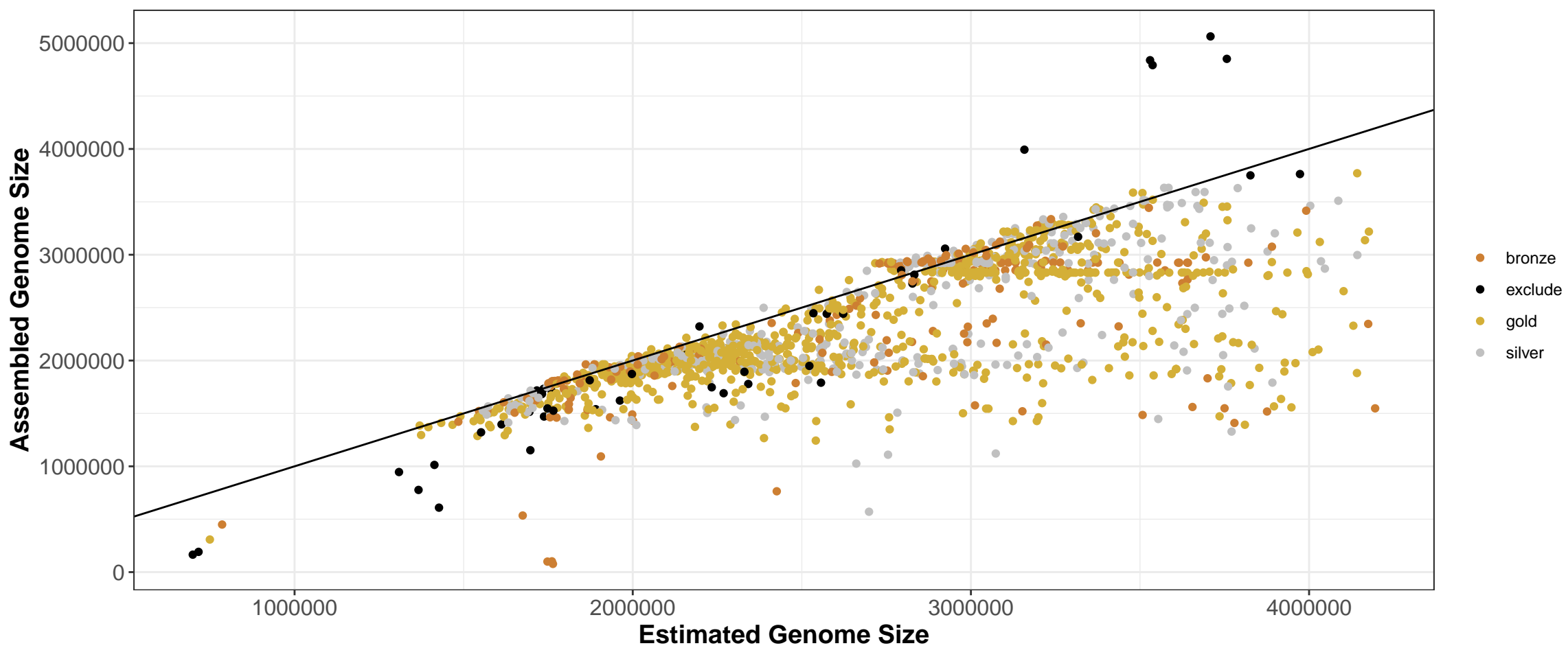

Supplement: FIG S3 [file mSystems.00190-20-sf003.pdf]

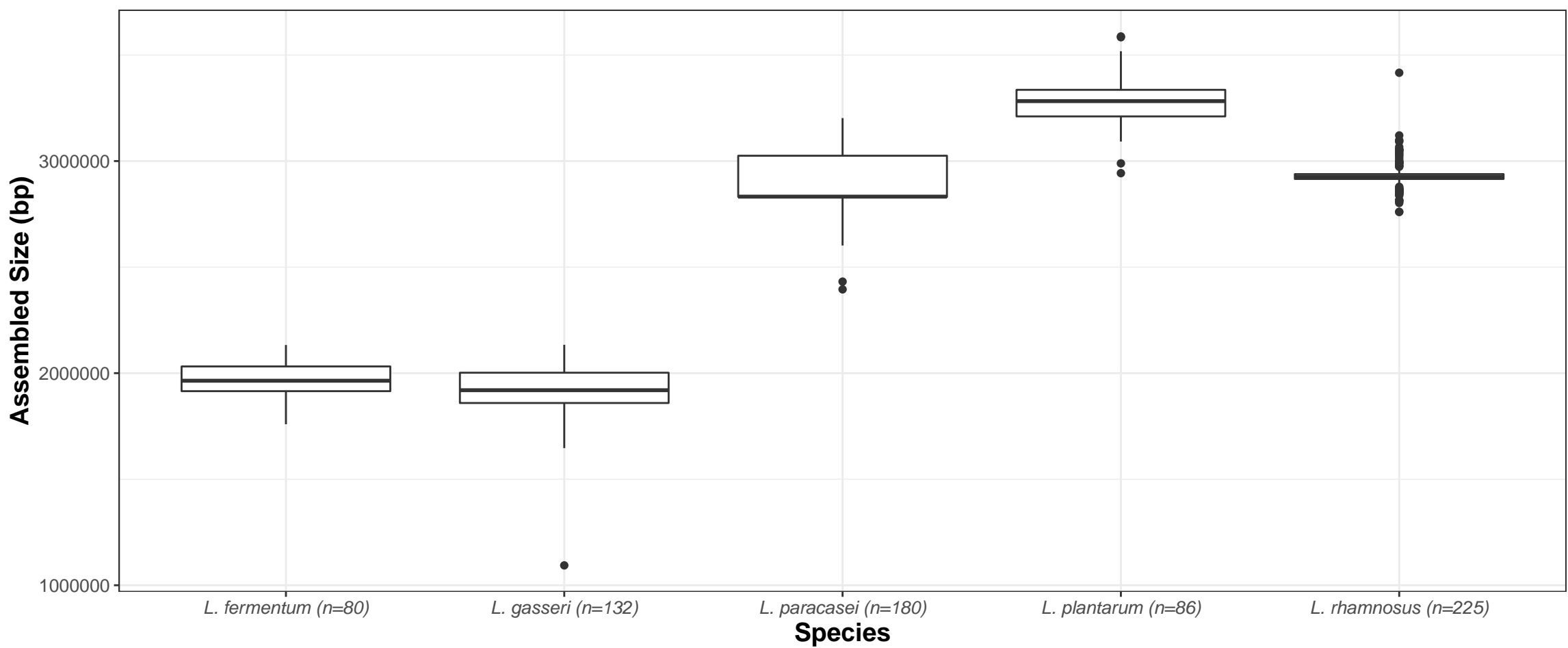

Supplement: FIG S4 [file mSystems.00190-20-sf004.pdf]
