# Supplementary material for: Bactopia: a Flexible Pipeline for Complete Analysis of Bacterial Genomes
Source: mSystems. 2020 Aug 4;5(4):e00190-20. doi: 10.1128/mSystems.00190-20 (PMC7406220; doi:10.1128/mSystems.00190-20)
Supplement: TABLE S1 [file mSystems.00190-20-st001.docx]

| **Sample** | **Exclusion Reason** |
| --- | --- |
| DRX061123 | Not processed, reason: Poor estimate of genome size |
| DRX061147 | Not processed, reason: Poor estimate of genome size |
| DRX143657 | Not processed, reason: Poor estimate of genome size |
| ERX034817 | Not processed, reason: Poor estimate of genome size |
| ERX1046386 | Not processed, reason: Low depth of sequencing |
| ERX1046387 | Not processed, reason: Low depth of sequencing |
| ERX1046388 | Not processed, reason: Low depth of sequencing |
| ERX1046389 | Not processed, reason: Low depth of sequencing |
| ERX1046390 | Failed to pass minimum cutoffs, reason: Low coverage (14.83x, expect >= 20x);Too many contigs (783, expect <= 500) |
| ERX1046391 | Failed to pass minimum cutoffs, reason: Too many contigs (631, expect <= 500) |
| ERX1046392 | Failed to pass minimum cutoffs, reason: Low coverage (11.54x, expect >= 20x);Too many contigs (755, expect <= 500) |
| ERX1046393 | Failed to pass minimum cutoffs, reason: Low coverage (17.59x, expect >= 20x);Too many contigs (544, expect <= 500) |
| ERX1046397 | Not processed, reason: Low depth of sequencing |
| ERX1046398 | Not processed, reason: Low depth of sequencing |
| ERX1046399 | Not processed, reason: Low depth of sequencing |
| ERX1046400 | Failed to pass minimum cutoffs, reason: Low coverage (11.97x, expect >= 20x);Too many contigs (810, expect <= 500) |
| ERX1046401 | Failed to pass minimum cutoffs, reason: Low coverage (18.17x, expect >= 20x);Too many contigs (559, expect <= 500) |
| ERX1046402 | Failed to pass minimum cutoffs, reason: Low coverage (13.30x, expect >= 20x);Too many contigs (857, expect <= 500) |
| ERX1046407 | Failed to pass minimum cutoffs, reason: Low coverage (18.63x, expect >= 20x) |
| ERX1046416 | Failed to pass minimum cutoffs, reason: Low coverage (19.20x, expect >= 20x) |
| ERX1275836 | Not processed, reason: Poor estimate of genome size |
| ERX1275870 | Not processed, reason: Low number of reads;Low depth of sequencing |
| ERX1275925 | Not processed, reason: Poor estimate of genome size |
| ERX1275959 | Not processed, reason: Low number of reads;Low depth of sequencing |
| ERX178660 | Not processed, reason: Poor estimate of genome size |
| ERX178661 | Failed to pass minimum cutoffs, reason: Too many contigs (1079, expect <= 500) |
| ERX178663 | Not processed, reason: Poor estimate of genome size |
| ERX2438180 | Not processed, reason: Poor estimate of genome size |
| ERX271964 | Failed to pass minimum cutoffs, reason: Too many contigs (900, expect <= 500) |
| ERX271983 | Failed to pass minimum cutoffs, reason: Too many contigs (515, expect <= 500) |
| ERX2866884 | Not processed, reason: Poor estimate of genome size |
| ERX303991 | Not processed, reason: Poor estimate of genome size |
| ERX359696 | Not processed, reason: Poor estimate of genome size |
| ERX359703 | Not processed, reason: Poor estimate of genome size |
| ERX359718 | Not processed, reason: Poor estimate of genome size |
| ERX359724 | Not processed, reason: Low number of reads;Low depth of sequencing |
| ERX359774 | Not processed, reason: Poor estimate of genome size |
| ERX359777 | Failed to pass minimum cutoffs, reason: Too many contigs (674, expect <= 500) |
| ERX399759 | Not processed, reason: Poor estimate of genome size |
| ERX450866 | Failed to pass minimum cutoffs, reason: Too many contigs (902, expect <= 500) |
| ERX450885 | Failed to pass minimum cutoffs, reason: Too many contigs (554, expect <= 500) |
| ERX529055 | Not processed, reason: Poor estimate of genome size |
| ERX529175 | Not processed, reason: Poor estimate of genome size |
| SRX059832 | Failed to pass minimum cutoffs, reason: Short read length (35.00bp, expect >= 49 bp) |
| SRX130889 | Not processed, reason: Low depth of sequencing |
| SRX130890 | Not processed, reason: Low depth of sequencing |
| SRX130892 | Failed to pass minimum cutoffs, reason: Too many contigs (618, expect <= 500) |
| SRX130895 | Failed to pass minimum cutoffs, reason: Too many contigs (1050, expect <= 500) |
| SRX130896 | Failed to pass minimum cutoffs, reason: Too many contigs (610, expect <= 500) |
| SRX130897 | Failed to pass minimum cutoffs, reason: Low coverage (18.31x, expect >= 20x);Too many contigs (940, expect <= 500) |
| SRX130898 | Not processed, reason: Low depth of sequencing |
| SRX130903 | Failed to pass minimum cutoffs, reason: Low coverage (17.02x, expect >= 20x);Too many contigs (677, expect <= 500) |
| SRX130904 | Failed to pass minimum cutoffs, reason: Too many contigs (719, expect <= 500) |
| SRX130910 | Failed to pass minimum cutoffs, reason: Low coverage (18.77x, expect >= 20x) |
| SRX130911 | Failed to pass minimum cutoffs, reason: Too many contigs (948, expect <= 500) |
| SRX130916 | Failed to pass minimum cutoffs, reason: Low coverage (16.95x, expect >= 20x) |
| SRX1490246 | Not processed, reason: Low number of reads |
| SRX1684187 | Not processed, reason: Poor estimate of genome size |
| SRX1684189 | Not processed, reason: Poor estimate of genome size |
| SRX1687039 | Not processed, reason: Poor estimate of genome size |
| SRX1842889 | Failed to pass minimum cutoffs, reason: Too many contigs (1016, expect <= 500) |
| SRX1949176 | Not processed, reason: Poor estimate of genome size |
| SRX1950180 | Failed to pass minimum cutoffs, reason: Short read length (35.00bp, expect >= 49 bp);Too many contigs (962, expect <= 500) |
| SRX1950181 | Failed to pass minimum cutoffs, reason: Short read length (35.00bp, expect >= 49 bp) |
| SRX1970179 | Not processed, reason: Poor estimate of genome size |
| SRX200229 | Not processed, reason: Poor estimate of genome size |
| SRX200230 | Not processed, reason: Poor estimate of genome size |
| SRX247326 | Not processed, reason: Poor estimate of genome size |
| SRX2582464 | Not processed, reason: Poor estimate of genome size |
| SRX2940498 | Not processed, reason: Poor estimate of genome size |
| SRX2940499 | Not processed, reason: Poor estimate of genome size |
| SRX3075577 | Not processed, reason: Poor estimate of genome size |
| SRX3146668 | Failed to pass minimum cutoffs, reason: Low coverage (18.90x, expect >= 20x);Too many contigs (784, expect <= 500) |
| SRX3155954 | Not processed, reason: Poor estimate of genome size |
| SRX318181 | Failed to pass minimum cutoffs, reason: Too many contigs (692, expect <= 500) |
| SRX377715 | Failed to pass minimum cutoffs, reason: Too many contigs (787, expect <= 500) |
| SRX422995 | Not processed, reason: Low number of reads;Low depth of sequencing |
| SRX4336892 | Not processed, reason: Poor estimate of genome size |
| SRX4526088 | Failed to pass minimum cutoffs, reason: Low coverage (16.08x, expect >= 20x) |
| SRX4526092 | Not processed, reason: Paired-end read count mismatch |
| SRX4997533 | Not processed, reason: Poor estimate of genome size |
| SRX5116733 | Not processed, reason: Poor estimate of genome size |
| SRX5116734 | Not processed, reason: Poor estimate of genome size |
| SRX5116735 | Not processed, reason: Poor estimate of genome size |
| SRX5116736 | Not processed, reason: Poor estimate of genome size |
| SRX5116737 | Not processed, reason: Poor estimate of genome size |
| SRX5395058 | Not processed, reason: Poor estimate of genome size |
| SRX5489807 | Not processed, reason: Poor estimate of genome size |
| SRX5992480 | Failed to pass minimum cutoffs, reason: Too many contigs (843, expect <= 500) |
| SRX5992985 | Failed to pass minimum cutoffs, reason: Too many contigs (1220, expect <= 500) |
| SRX5992988 | Failed to pass minimum cutoffs, reason: Low coverage (9.19x, expect >= 20x);Too many contigs (1440, expect <= 500) |
| SRX5992989 | Not processed, reason: Poor estimate of genome size |
| SRX6454026 | Failed to pass minimum cutoffs, reason: Too many contigs (533, expect <= 500) |
| SRX6454030 | Failed to pass minimum cutoffs, reason: Too many contigs (796, expect <= 500) |
| SRX6454035 | Failed to pass minimum cutoffs, reason: Too many contigs (687, expect <= 500) |
| SRX6454040 | Failed to pass minimum cutoffs, reason: Too many contigs (792, expect <= 500) |
| SRX6454043 | Failed to pass minimum cutoffs, reason: Too many contigs (801, expect <= 500) |
| SRX6959882 | Failed to pass minimum cutoffs, reason: Low coverage (16.59x, expect >= 20x);Too many contigs (855, expect <= 500) |
| SRX7004909 | Failed to pass minimum cutoffs, reason: Too many contigs (999, expect <= 500) |
| SRX761992 | Failed to pass minimum cutoffs, reason: Low coverage (18.30x, expect >= 20x) |
| SRX762105 | Failed to pass minimum cutoffs, reason: Low coverage (15.33x, expect >= 20x);Too many contigs (560, expect <= 500) |
| SRX762278 | Not processed, reason: Poor estimate of genome size |
| SRX762279 | Failed to pass minimum cutoffs, reason: Low coverage (16.51x, expect >= 20x) |
| SRX762385 | Failed to pass minimum cutoffs, reason: Too many contigs (1132, expect <= 500) |
| SRX762507 | Failed to pass minimum cutoffs, reason: Low coverage (16.75x, expect >= 20x) |
| SRX762687 | Failed to pass minimum cutoffs, reason: Low coverage (19.54x, expect >= 20x) |

## 
