# Supplementary material for: Bactopia: a Flexible Pipeline for Complete Analysis of Bacterial Genomes
Source: mSystems. 2020 Aug 4;5(4):e00190-20. doi: 10.1128/mSystems.00190-20 (PMC7406220; doi:10.1128/mSystems.00190-20)
Supplement: TABLE S2 [file mSystems.00190-20-st002.docx]

| **sample** | **sra** | **gtdb** |
| --- | --- | --- |
| ERX2000473 | Lactobacillus jensenii | Actinomyces viscosus |
| ERX1275832 | Lactobacillus rhamnosus | Aerococcus urinae |
| ERX1275921 | Lactobacillus rhamnosus | Aerococcus urinae |
| ERX2000484 | Lactobacillus iners | Bifidobacterium vaginale |
| ERX1275866 | Lactobacillus rhamnosus | Bifidobacterium vaginale |
| ERX1275955 | Lactobacillus rhamnosus | Bifidobacterium vaginale |
| ERX1275856 | Lactobacillus fermentum | Campylobacter ureolyticus |
| ERX1275945 | Lactobacillus fermentum | Campylobacter ureolyticus |
| SRX301579 | Lactobacillus catenefornis DSM 20559 | Eggerthia catenaformis |
| ERX2000470 | Lactobacillus jensenii | Facklamia hominis |
| ERX034808 | Lactobacillus sp. | KLE1615 sp900066985 |
| SRX2118809 | Lactobacillus rogosae | Lachnospira rogosae |
| ERX1275881 | Lactobacillus jensenii | Microbacterium sp001595495 |
| ERX1275970 | Lactobacillus jensenii | Microbacterium sp001595495 |
| SRX963052 | Lactobacillus sp. HMSC12B03 | Neisseria bacilliformis |
| ERX1275835 | Lactobacillus iners | Pseudoglutamicibacter cumminsii |
| ERX1275924 | Lactobacillus iners | Pseudoglutamicibacter cumminsii |
| ERX178670 | Lactobacillus casei | Staphylococcus epidermidis |
| SRX244574 | Lactobacillus gasseri ADL-351 | Streptococcus agalactiae |
| ERX1275883 | Lactobacillus sp. | Streptococcus agalactiae |
| ERX1275972 | Lactobacillus sp. | Streptococcus agalactiae |
| ERX2000463 | Lactobacillus johnsonii | Streptococcus parasanguinis |
| ERX2000481 | Lactobacillus crispatus | Streptococcus pasteurianus |
| ERX3310397 | Lactobacillus acetotolerans | Streptococcus pneumoniae |
| ERX3310420 | Lactobacillus acidophilus | Streptococcus pneumoniae |
| ERX3310369 | Lactobacillus agilis | Streptococcus pneumoniae |
| ERX3310458 | Lactobacillus alimentarius | Streptococcus pneumoniae |
| ERX3310319 | Lactobacillus amylophilus | Streptococcus pneumoniae |
| ERX3310443 | Lactobacillus amylovorus | Streptococcus pneumoniae |
| ERX3310479 | Lactobacillus animalis | Streptococcus pneumoniae |
| ERX3310410 | Lactobacillus aviarius | Streptococcus pneumoniae |
| ERX3310408 | Lactobacillus bifermentans | Streptococcus pneumoniae |
| ERX3310303 | Lactobacillus brevis | Streptococcus pneumoniae |
| ERX3310449 | Lactobacillus buchneri | Streptococcus pneumoniae |
| ERX3310425 | Lactobacillus casei | Streptococcus pneumoniae |
| ERX3310459 | Lactobacillus coryniformis | Streptococcus pneumoniae |
| ERX3310264 | Lactobacillus delbrueckii subsp. bulgaricus | Streptococcus pneumoniae |
| ERX3310316 | Lactobacillus delbrueckii | Streptococcus pneumoniae |
| ERX3310336 | Lactobacillus farciminis | Streptococcus pneumoniae |
| ERX3310280 | Lactobacillus fermentum | Streptococcus pneumoniae |
| ERX3310372 | Lactobacillus fructivorans | Streptococcus pneumoniae |
| ERX3310460 | Lactobacillus helveticus | Streptococcus pneumoniae |
| ERX3310328 | Lactobacillus hilgardii | Streptococcus pneumoniae |
| ERX3310291 | Lactobacillus mali | Streptococcus pneumoniae |
| ERX3310432 | Lactobacillus oris | Streptococcus pneumoniae |
| ERX3310305 | Lactobacillus paracasei | Streptococcus pneumoniae |
| ERX3310374 | Lactobacillus pentosus | Streptococcus pneumoniae |
| ERX3310445 | Lactobacillus plantarum | Streptococcus pneumoniae |
| ERX3310452 | Lactobacillus ruminis | Streptococcus pneumoniae |
| ERX3310353 | Lactobacillus sakei | Streptococcus pneumoniae |
| ERX3310387 | Lactobacillus sakei L45 | Streptococcus pneumoniae |
| ERX3310490 | Lactobacillus salivarius | Streptococcus pneumoniae |
| ERX3310309 | Lactobacillus sanfranciscensis | Streptococcus pneumoniae |
| ERX3310430 | Lactobacillus sharpeae | Streptococcus pneumoniae |
| ERX3310388 | Lactobacillus sp. 30A | Streptococcus pneumoniae |
| ERX3310483 | Lactobacillus vaginalis | Streptococcus pneumoniae |
| ERX3310456 | Lactobacillus vermiforme | Streptococcus pneumoniae |
| ERX2000443 | Lactobacillus gasseri | Winkia sp002849225 |
