# Supplementary material for: Bactopia: a Flexible Pipeline for Complete Analysis of Bacterial Genomes
Source: mSystems. 2020 Aug 4;5(4):e00190-20. doi: 10.1128/mSystems.00190-20 (PMC7406220; doi:10.1128/mSystems.00190-20)
Supplement: FIG S1 [file mSystems.00190-20-sf001.pdf]

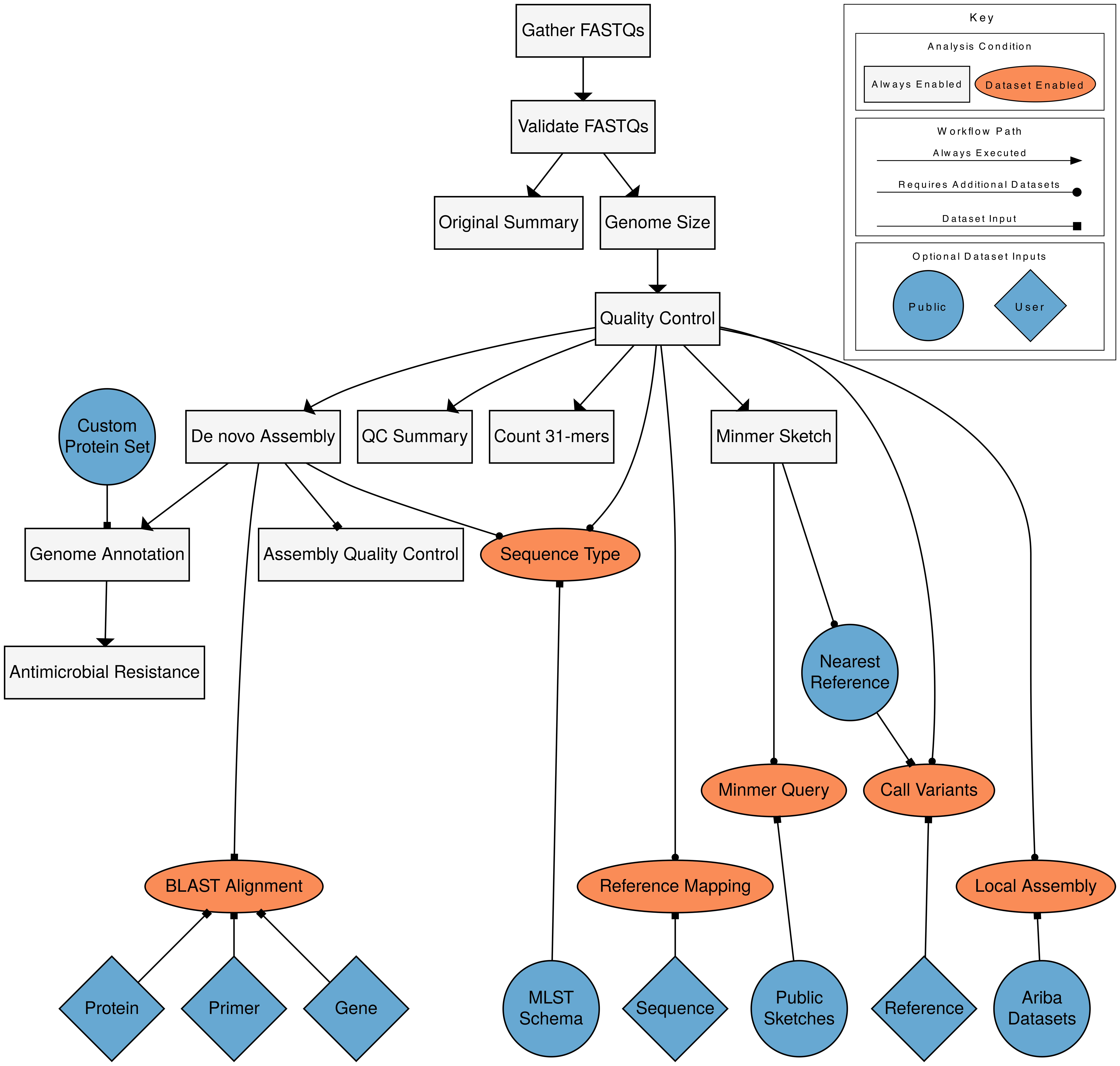

Gather FASTQs
Validate FASTQs
Original Summary
Genome Size
Quality Control
Custom Protein Set
De novo Assembly
QC Summary
Count 31-mers
Minmer Sketch
Genome Annotation
Assembly Quality Control
Sequence Type
Antimicrobial Resistance
Nearest Reference
Minmer Query
Call Variants
BLAST Alignment
Reference Mapping
Local Assembly
Protein
Primer
Gene
MLST Schema
Sequence
Public Sketches
Reference
Ariba Datasets
Key
Analysis Condition
Always Enabled
Dataset Enabled
Workflow Path
Always Executed
Requires Additional Datasets
Dataset Input
Optional Dataset Inputs
Public
User
